# Challenges around Child-Feeding Practices with ‘Comida Chatarra’: A Qualitative Study to Understand the Role of Sociocultural Factors in Caregiver Feeding Decisions

**DOI:** 10.3390/nu15061317

**Published:** 2023-03-07

**Authors:** Florence L. Théodore, Anabelle Bonvecchio, Ana Lilia Lozada Tequeanes, Rocío Alvarado, Armando García-Guerra, María Angeles Villanueva Borbolla, Mauro Brero

**Affiliations:** 1 Centro de Investigación en Nutrición y Salud (CINyS), Instituto Nacional de Salud Pública, Avenida Universidad 655, Santa María Ahuacatitlán, Cuernavaca 62100, Mexico; 2 Unicef México, Ciudad de México 11000, Mexico

**Keywords:** ultra-processed products, emic perspective, qualitative method, phenomenology

## Abstract

A massive incorporation of ultra-processed products into young children’s diets worldwide and in Mexico has been documented. The aim of this study is to understand the role of sociocultural factors in principal caregivers’ decisions to give a type of ultra-processed food to children under age five, called ‘comida chatarra’ (‘junk food’ in English), usually includes sugar-sweetened beverages, sweet and salty snacks, and sweet breakfast cereals. We conducted a descriptive, observational qualitative study. The research was conducted in urban and rural communities in two Mexican states. Twenty-four principal caregivers were equally distributed between the two states and types of communities. They were interviewed in person. Phenomenology underpinned this study. Results highlight the preponderant role of culture in food choices and feeding practices with junk food. Local culture influences child-feeding with ultra-processed products through social norms, knowledge, or socially constructed attitudes. These social norms, built in the context of abundant ultra-processed products and omnipresent marketing, ‘justify’ children’s consumption of junk food. They acquire these products from the principal caregivers, family members, and neighbors, among others, who reward and pamper them. These actors also define what amount (small amounts) and when (after meals as snacks) children are given these products. Cultural factors must be considered in the development of effective public policies and programs that aim to change the culture around ultra-processed products among children and avoid their consumption.

## 1. Introduction

For more than two decades, the massive incorporation of ultra-processed products (UPPs) into the diets of adults and young children has been widely documented worldwide and especially in Latin America [1,2]. These artificial products are unhealthy due to the way they are manufactured and their high content of added sugar, salt, and/or fat [3]. UPPs are generally directed at children and adolescents. They include sugar-sweetened beverages, sweet and salty snacks, processed meat, and sweet breakfast cereals. UPPs have been associated with a large spectrum of preventable communicable diseases [4]. The high level of UPP consumption is the result of political and economic processes related to liberalism, free trade agreements [5], and tourism development [6] that shaped the obesogenic environment [7] under which UPP intake is encouraged through powerful marketing strategies aimed at positioning these products as widely available and desirable [8].

Rural and urban landscapes in Mexico with the highest consumption of UPPs are described as ‘food swamps’ [9,10], a geographic metaphor for the inundation of unhealthy products in communities with healthy food options [11]. Food swamps predict obesity rates [12]. In Mexico, UPPs are extensively advertised in public spaces, including streets [13], grocery stores, TV [14], social media [15], and inside and around schools [16,17]. Unlike Uruguay [18], the terminology for UPPs is unknown for the Mexican population, and the closest term is ‘comida chatarra’ (‘junk food’ in English) [19]. In this article, ‘comida chatarra’ or ‘chatarra’ will refer to the participants’ own use, whereas the term “UPPs” will refer to the way in which these unhealthy products are named according to the NOVA classification [20].

Research on UPPs has focused on measuring purchases [21] or intake [22] among adults and children, as well as associated harms to health [4,23,24]. Other studies have analyzed conceptualizations of UPPs [25,26,27] and identified the drivers and facilitators of their intake [19,28]. This evidence is essential to guide the development of public policies and educational programs aimed at preventing obesity, a major public health issue in Mexico [29,30].

However, little is known about caregivers’ experiences emerging in this food swamp when providing food to children under age five, where antagonistic narratives about UPPs coexist in terms of their content and healthiness, which is important to caregivers worldwide [26,31]. The consumption of UPPs is reported from early childhood [32,33,34], a life stage considered particularly ‘vulnerable’ as malnutrition can lead to irreversible consequences for growth and development [35,36], and the food habits adopted can endure for life.

Therefore, it is essential to understand which factors and processes influence principal caregivers’ (PCs) decisions to give UPPs to children under age five. It is important to study this from their perspective and from different theoretical frameworks, such as the theory of planned behavior, food choice decision framework, or the behavior change wheel, among others [37,38,39].

This article focuses on the role of sociocultural factors in PCs’ experiences and decisions to give UPPs to children under age five, analyzing the meaning of ‘chatarra’ and the different decisions made by caregivers in Mexico from rural and urban landscapes. Culture is understood as a set of traditions, lifestyles, knowledge, beliefs, and norms shared by the members of a society, which are socially acquired [40]. Food is shaped by culture [41], but culture also shapes food choices and the willingness to incorporate new foods [42,43].

We aimed to understand how PCs navigate the food swamp, as well as their experience with ‘chatarra’ and feeding practices of children under age five by analyzing their practices, beliefs and knowledge, attitudes and social norms, and the way they give meaning to their actions.

This study aimed to inform the design of a mHealth strategy to prevent malnutrition among children under age five and promote behavior change towards a ‘healthy’ lifestyle by sending short message services (SMS) about nutrition and health to PCs living in vulnerable economic situations.

## 2. Materials and Methods

### 2.1. Research Design

Our study was descriptive, observational, and qualitative. We conducted 24 in-depth face-to-face interviews with PCs.

### 2.2. Theoretical Framework

Phenomenology underpinned this exploratory qualitative research study, which focused on lifeworld study and the subjective consciousness and experience of people built through daily life interactions and dialogue among individuals [44]. This framework guided how we explored the subjective lived experience of PCs and how we interpreted their perceptions and feeding practices related to ‘comida chatarra’, a type of UPP.

### 2.3. Setting, Participants, and Purposive Sampling

We conducted our research in urban and rural communities of two states (in the south and center regions of Mexico). The first was the state of Yucatan, located in the peninsula of Yucatan, with predominant Mayan culture, which has undergone great changes from traditional to globalized diet [32]. The second was the state of Morelos, with easy access to big cities, located about eighty kilometers from Mexico City, one of the most populated cities in the world.

States were selected according to the implementation feasibility of the project.

In-depth face-to-face interviews with PCs were equally distributed between the two states in urban and rural communities. UPPs were one of the general health and nutrition topics explored.

Our sampling was purposive [45]. Considering that experiences with infant feeding could depend on other people, on the state, the type of locality (urban and rural), and whether women were first-time mothers or not, we introduced this diversity when constructing our sample. Inclusion criteria were being a PC of children aged 24–59 months (although we explored feeding experience from birth) in charge of their daily feeding and care, having access to a functional cell phone, and being a beneficiary of the federal social protection conditional cash transfer program named Prospera [46] due to economic marginalization defined by the lack of education, housing and availability of goods.

Being a PC of a child with a disease involving a particular diet was the only exclusion criterium because feeding experiences might have been different.

The research team recruited participants through primary health care centers based on a list of caregivers who met the study’s inclusion criteria.

### 2.4. Data Collection

Topics explored with PCs in the in-depth interviews included: family dynamics around food and UPPs consumption (including industrial sugar beverages), practices, knowledge (especially harm and benefits to health), attitudes, and social norms related to the consumption of UPPs by infants and young children. To study the concept of UPPS, we explored topics with the participants using the words ‘chatarra’ and ‘comida chatarra’.

Prior to the interview, informants answered a sociodemographic questionnaire (age, level of education, number of children, among others).

In each state, two teams of three junior researchers with master’s degrees in public health (5 females and 1 male) completed a three-day training and piloted the instrument. The fieldwork was conducted for two weeks in October (Yucatán) and two weeks in November 2019 (Morelos). A senior social science researcher designed the qualitative study, supervised the fieldwork, and analyzed and interpreted data. Our fieldwork was conducted before the approval of the new nutritional warning labels (NOM-051) in January 2020, which allows consumers to quickly identify healthy and unhealthy products [47].

All interviews were conducted in Spanish and lasted between 45 and 80 min. We translated the selected testimonials for this article into English.

### 2.5. Analysis

In-depth interviews were audio recorded and transcribed verbatim and verified for quality.

To interpret the in-depth interviews, the team followed a systematic procedure: (1) Categories and dimensions were identified using the initial in-depth interview guide based on phenomenology (gathering data on experiences) and the research question; (2) Emerging categories were included in the coding tree after a team discussion (Figure S1); (3) Encoding was performed with NVivo^®^ software and results were interpreted from interference and contextualization of the testimonials [48]. The six junior researchers coded the interviews, and the senior researcher supervised the process and organized regular meetings to ensure a standardized process. Using grounded theory and comparative methods [49], a junior and senior researcher (one per state) interpreted the data and were in constant communication to guarantee validity.

## 3. Results

### 3.1. PCs’ Characteristics

The average age of the sample was 34.6 years for Yucatan and 32.2 years for Morelos. Women from Morelos had a slightly higher educational level and a number of children (more than three children per PC) (Table 1). Our sampling consisted only of the children’s mothers. The sociodemographic information of each of the 24 participants is presented in Table S1.

Despite cultural specificities, we detected little differences between PC’s experiences with UPPs in Yucatan and Morelos. Therefore, the information is presented without distinguishing between the states.

### 3.2. UPP Availability and Child-Feeding Practices

The PCs reported obtaining food from markets, supermarkets, and local stores (including greengrocers and poultry stores) and from backyards and fruit trees on a regular basis. All PCs mentioned a high availability of ‘chatarra’ in their neighborhood, thanks to an extensive network of street stalls (especially around schools) and grocery stores, where children usually hung out. In urban settings, PCs mentioned a greater diversity of UPPs.

Throughout the day, PCs routinely offered children a variety of ‘chatarra’. The most mentioned type of ‘chatarra’ was cookies, added sugar yogurts, drinkable yogurts, candies, gum, chips, ‘chicharrón de harina’ (fried Mexican snacks made with flour and salt), and pizza, among others. In the urban communities of both states, PCs also mentioned offering flan (a Mexican dessert), a high number of chip brands, pastries, and powdered chocolate in drinks. Sweet breakfast cereals were mentioned in the urban and rural landscapes of both states and in the rural communities of Morelos.

In this context of high availability, the PCs saw children’s consumption of ‘chatarra’ as “normal” nowadays and said that it had been normalized over the years:

*“(...) in my family you see it as something normal [consumption of ‘chatarra’ by children], something [that is] part of their daily life.”*

(#22)

Finally, ‘chatarra’ was also associated with celebrations and conviviality.

### 3.3. PC’s Knowledge and Attitudes of ‘Chatarra’

PCs are immersed in a universe where conflicting information about UPPs coexists, as presented below. It is important to understand knowledge and attitudes toward ‘chatarra’ to understand the way PCs manage UPPs with children.

#### 3.3.1. Definition of ‘Chatarra’

PCs mentioned a small number of products that they considered as ‘chatarra’: chips, ‘chicharron de harina’ (in Spanish), candies, store-bought cookies, and cola soda. Most ‘chatarra’ products are packaged. Urban PCs had a broader conception of ‘chatarra’, including foreign foods such as hamburgers, hotdogs, pizza, or instant food. One PC even mentioned industrial baby food jars and yogurts. However, generally, yogurts (those with added flavors and sugars) are considered healthy food by PCs.

PCs noted the following features of ‘chatarra’: unnatural because it is made of chemicals and preservatives, with high content of sugar and/or fat, and no nutrients or vitamins associated with good health and growth among young children.

*“Among them is the soft drink […] because it has a lot of chemicals […] they are made with artificial flavors.”*

(#8)

However, we did identify inconsistencies in the PCs’ own storytelling; products that should belong to ‘chatarra’, according to the previously mentioned characteristics, are not always considered as such by PCs. For example, orange-flavored soda or industrial juice or chocolate powder because they supposedly contain ‘vitamins,’ as announced in the brand slogans:

*“Because it is made from natural fruit [referring to an industrial juice] […] it is thick, you can see […] there are others that come that way, they are not very watery […] there is a XX [name of a juice brand] that comes in a bottle […] well, it is good because I think that to give something to children, you have to read [the label] on the product [...] if it is nutritious for them [...] well I see that it says 100% natural fruit, so it has this [...].”*

(#8)

Despite being products that are high in sodium, sugar, and chemicals, a wide range of UPPs are not considered ‘chatarra’ by PCs, such as processed meats and sausages, which are widely consumed by children for being “a cheap nutritive food”.

In brief, PCs’ knowledge of these product characteristics and their health effects are based on a variety of sources that are not always aligned with nutritional recommendations, and this guides their navigation in the food swamp.

#### 3.3.2. Beliefs about Health Effects

Knowledge about the health issues that can be caused by ‘chatarra’ consumption came from many sources (health centers, schools, own experiences, the folk culture).

PCs expressed that ‘chatarra’ do not nourish children, unlike vegetables, fruits, and meat (including processed meats and sausages or tortillas, which are sources of nutrients and vitamins necessary to prevent diseases (urban landscapes) and to guarantee healthy growth (rural landscapes).

*“I say that the cookies don’t have vitamins, the chips don’t either…it’s not exactly like the meat, the chicken, the pork, or the vitamin in the stock.”*

(#5)

*“Well, because they do not include let’s say vitamins” (…) ‘Chatarra’ are tasty but nothing else because they harm his body (…) I consider that if he eats a lot of those things, he is going to gain too much weight and he has no defenses” (…) he is not going to have his defenses, for example, if he gets the flu, the flu will take a long time, because he has no defenses strong [enough] to, to fight it.”*

(#9)

PCs also associated these ‘chatarra’ with indigestion (‘empacho’, a Latin-American folk illness), grounded on the idea that children’s stomachs are not ready for certain junk food:

*“Well, infections [are caused by junk food], because then the children eat a lot of things. They say that they are stuffed. We bring them to the doctor, and they tell us it’s an infection. Then, one rubs their stomach, or we bring them to cure ‘empacho’ and someone puts oil on them. When children eat junk food, well, they smell very bad, because all of this is as if we had a hose and that when the water passes or a leaf gets stuck, it no longer passes, like our body.”*

(#13)

PCs considered that not all these ‘chatarra’ had the same harmful potential, ranking them according to the degree of assessed damage. PCs perceived chips and soda as very harmful, while they considered that store-bought cookies “do not harm and do not benefit” (#4). According to PCs, in addition to the chemicals, soda has a high-sugar content responsible for diabetes. Sugar seemed to be an ingredient feared by PCs and identified by them in sweets, juices, and soda, among others, creating a kind of Saccharophobia towards sweet UPPs. Some PCs included yogurts as they contain milk, and although they also have preservatives, PCs consider that it is much better to give children yogurt than chips (#17).

Finally, most PCs associated ‘chatarra’ consumption with obesity and diabetes.

*“Sometimes we give children a lot of sweets, chips, and that’s why this happens [obesity], a lot of sugar, then there are many who are already obese or have diabetes.”*

(#18)

Although some PCs commented that they strive to avoid children’s consumption of those very harmful ‘chatarra’ (soft drinks, chips), they emphasized their lack of control because of two main factors: children’s taste preferences and the role modeling of family members (elder siblings, parents, grandparents), friends, or neighbors they see eating ‘chatarra’. Eating and drinking chatarra throughout the day has become a social norm in all age groups of rural and urban communities of these two states, normalizing snacking among adults and children.

However, there was a moral judgment of ‘laziness’ towards PCs who gave these ‘chatarra’ to children instead of nutritious food,

*“Because they are too lazy to cut the fruit and they don’t do it, they don’t give it. (…) It is a question of convenience.”*

(#18)

#### 3.3.3. Hedonist Domain

Regardless of their knowledge about the damage of ‘chatarra’ to health, PCs assimilated them as a ‘craving’ or a ‘treat’ for children to be given in addition to food considered as nutritious.

*“For me it’s the same [chatarra and treats] (...) for me it’s chips, candy, chocolate bars, soft drinks” (…) “Well, I say that candy or chips would be eaten as a snack, right? As something that children like, but later they don’t eat, because that’s not correct.”*

(#18)

By establishing it as a ‘craving’ and a snack, a very general idea in both states, PCs differentiated it from the home food considered nutritious. From what was reported by PCs, ‘chatarras’ function is purely hedonistic but can also be problematic when children only want to eat it, ignore homemade food, or get sick.

*“It’s only for taste [...] it’s bad because they don’t eat well, that’s when they get sick, they get sick, they can’t stand the illness anymore, diarrhea, a fever, they throw them away quickly because they’re not fed enough.”*

(#8)

An emerging theme of this study is the tension experienced by PCs between two principles that structure their actions towards child-feeding that placed them in a difficult situation with ‘chatarra’. On the one hand, they seek to ensure children’s health and growth, and on the other hand, they attach importance to pleasing their children by satisfying their cravings and rewarding them with ‘chatarra’, which is stimulated by an obesogenic environment:

*“When leaving the classroom, if they behaved well, there is no complaint, this “mom, I want a sweet” and I buy it for them.”*

(#2)

*“(...) his dad takes him to the grocery store when he behaves well, I’m going to buy you some chips XX because you behaved well, you were quiet.”*

(#23)

In summary, according to PCs, ‘chatarra’ is considered a craving, treat, or prize that can also be harmful to children’s health. As a caregiver or a mother, the way to ‘treat’ little children is with ‘chatarra’, a social norm that promotes its consumption by children.

Although economic constraints constitute a protective factor against UPP consumption, families’ poverty and lack of money to buy these products to fulfill their children’s cravings can generate frustration among PCs.

*“Chips, I usually do not buy it, because of the economy. It costs twelve pesos (..) sometimes I only have 200 pesos left to buy food for the day and I must pay for breakfast and for the children who go to school, and I must see how to make it so I can’t.”*

(#10)

*“[...] sometimes they sell very expensive things and I tell them that I don’t have that money, and I can only give them two to five pesos because I don’t have enough money.”*

(#12)

#### 3.3.4. Towards Reconciliation of Health and Hedonic Principles

We identified two main ideas in the PCs’ narratives that allowed them to continue giving ‘chatarra’ to children, including those considered to be the most harmful. First, PCs thought that giving them occasionally or in what they considered to be small amounts (called ‘probaditas’ in Spanish) of UPPs mitigated the potentially harmful effects.

*“I don’t give him much, yes, he loves cola soda, but I don’t give him much. Well, because I say, it is going to hurt him, because I was told that cola soda is harmful [LAUGHS] Because it has a lot of sugar. When my children were very young, I was told that they’d become diabetic when they were 15 years old if I would have given them soda every day. So, I thought it was better not to give it to them always. Just a little, they can have a small taste.”*

(#20)

From this PC’s narrative, offering ‘probaditas’ (small portions of UPPs) allowed PCs to reconcile young children’s pleasure and health, two a priori incompatible aims.

Second, offering ‘chatarra’ to children followed certain rules, such as not giving it to them before meals, avoiding displacing homemade meals, and guaranteeing necessary requirements for children’s growth and health.

We identified more strategies implemented by PCs to control the amount of ‘chatarra’ consumed by children to mitigate its potential damage.

PCs offered an option of ‘chatarra’ to children that they considered to be less harmful, not necessarily based on nutritional knowledge of the product, for example, the choice of orange soda instead of cola soda. Another strategy consisted of reducing the amount of ‘chatarra’ given to children by mixing it with foods and drinks considered healthy, such as mixing cola soda with water or sugary cereals with those that are not. Sharing a single package of chips among several children or giving them cookies or candies little by little were other examples given by PCs to limit children’s ‘chatarra’ consumption. Another strategy mentioned was aimed at avoiding stimulating the children’s desire to consume chips or sodas, asking siblings or the father to be discrete when eating these ‘chatarra’.

## 4. Discussion

This article highlights the preponderant role of culture in food choices and feeding practices in two Mexican states. We focused on a type of UPP referred to as ‘comida chatarra’, which is broadly incorporated into children’s diets in part thanks to powerful marketing strategies. PCs and environmental factors contribute to the normalization of child-feeding with ‘comida chatarra’ through a series of practices, experiences, and meanings linked to sociability, affection, and maternity. This role of culture in food choices and feeding practices has been previously documented elsewhere [50,51] in Mexico.

As far as we are aware, this is the first study that investigates meaning and mediations related to some UPPs (‘chatarra’) in the context of children’s feeding practices and behaviors through PC’s experiences. The study underlined the way local culture influences children’s feeding with UPPs through social norms, knowledge, or socially constructed attitudes. Regarding child-feeding with ‘chatarra’, we identified a series of social norms built in the context of abundant UPPs and the omnipresence of their marketing. In a way, these social norms ‘authorize’ children to eat junk food, obtaining it from PCs, family members, and neighbors, among others, with the purpose of rewarding and pampering them, as previously documented [52]. Social norms also define in what amount (small amounts) and when (after a homemade meal as a snack) to give these products. Through an approach focused on the study of daily life, we identified five findings that represent major worldwide public health concerns.

First, in our research, the universe of products considered as ‘chatarra’ by PCs is varied but does not include all UPPs, leaving out some that are widely consumed by children in Mexico and considered nutritious food (e.g., processed meat, industrial baby food jar), an important public health issue if we consider the associated health harms. As also reported in Uruguay, not all UPPs were considered unhealthy and detrimental to children’s health and growth by PCs [26], which was probably due to the lack of a simple and comprehensible food labeling system in both countries at the time of the studies, as well as misconceptions caused by marketing. From a public health perspective, actions for raising the population’s awareness about the large range of UPPs that are not only limited to a few numbers of ‘chatarra’ products are urgently required. In this context, food labeling acquires relevance as an instrument to support healthy choices among the population.

Second, PCs reported a wide availability of ‘chatarra’. Our data suggest that child-feeding with ‘chatarra’ is promoted by the food environment, prompted by the wide availability of UPP in close proximity, which has also been documented worldwide [53,54,55]. Moreover, advertising messages form positive perceptions of UPPs among PC. Omnipresent networks of stores constantly encourage UPP consumption among children and their provision by an immediate circle of acquaintances [56]. This routine consumption of ‘chatarra’ by children in Mexico contrasts with what was documented in Brazil, where it is conceptualized as breaking the routine that is based on home-style food [57]. We do hypothesize that commercial strategies of food corporations aimed at converting ‘chatarra’ into highly available, accessible, desirable, and hyper-palatable products have facilitated its incorporation into the population’s daily life and culture (including children). These commercial strategies are particularly effective in low- and middle-income countries, most of which share limited governance [58] that favors a significant permeation of companies in all aspects of society (legal, economic, social, and cultural) [59]. This makes it difficult to adopt policies to control corporate practices [60]. High availability of ‘chatarra’ results in its continued consumption by children. The main limitations for ‘chatarra’ consumption are economic constraints, while in other countries, junk food consumption by children has been associated with “socioeconomic adversity and family dysfunction that offspring distress” [61].

Third, PCs’ knowledge of product characteristics and health effects influences their navigation in the food swamp. They rank ‘chatarra’ according to their perceived outcomes on children’s growth and health based on a variety of sources that are not always aligned with nutritional science. The same situation was reported in Uruguay, where not all junk food was considered unhealthy and harmful [26].

Fourth, the incorporation of chatarra’ in children’s feeding practices may have been allowed by the cultural practice of ‘probaditas’ (small portions), a generalized strategy used to introduce babies to their first solid foods in Mexican culture [62,63]. ‘Probaditas’ are aimed at preparing the stomach to get used to new foods gradually, preventing ‘empacho’ (indigestion) [31]. With UPPs, ‘probaditas’ are extended to a later age and are used to avoid ‘empachos’, and, nowadays, obesity and diabetes. The second cultural conception based on a previously documented principle in relation to school snacks is that as long as children’s nutritional requirements are met, giving them junk food does not matter [64]. Finally, just like in Uruguay [26], ‘chatarra’ is placed in the hedonic domain. According to the traditional Mexican motherhood model, in Mexico, PCs have to satisfy a craving, where the child’s food preference is an important driver of infant feeding choices [31]. In countries where child-feeding with junk food is a public health issue, it would be important to identify the cultural anchors that may have fostered its consumption among children.

Fifth, children and the Mexican population, in general, have incorporated ‘chatarra’ in their day-to-day routine as documented [21,65] and have created positive meanings because its consumption is associated with affection, celebration, and conviviality, as documented in other countries [18,66]. The incorporation of junk food in family diets undoubtedly shapes children’s preferences and intake patterns [67].

In short, ‘chatarra,’ a type of UPP in Mexico and possibly in other countries, has penetrated daily life and culture since childhood through different processes. It is important to understand this cultural background to inform the design of public policy aimed at discouraging child-feeding with junk food. Nevertheless, the challenge is not only to avoid diseases associated with UPP consumption among children but to delay their initiation, considering their hyper-palatability and the subsequent conditioning to this type of flavors and food.

This study demonstrates the presence of various social norms that drive child-feeding with UPPs, which have evolved in the context of massive supply and marketing. Finally, we identified spaces (e.g., groceries) that stimulate the consumption of products aimed at children (sweets, candies), probably because they are in full view. Changes in social norms towards UPPs are urgently needed to reverse their favorable image, as was performed with tobacco over many years.

Finally, it seems to us that there is a need for greater control of the presentation of food and beverages aimed at children within the points of sale, which should not be visible to children.

The main limitation of this research is that UPPs were not the only topic explored in this study, which made it impossible to go deeper into the topic. In addition, the study predates the implementation of the new nutritional warning labels, and it would be interesting to explore its possible effects on child-feeding with UPPs. However, the information generated is rich and can inform possible strategies to minimize UPPs in children’s feeding, such as awareness campaigns and regulation of marketing aimed at children in stores, among others.

## 5. Conclusions

The influence of local knowledge, values, habits, and social norms is very relevant in shaping the decision of PCs about child-feeding and must be considered for the development of effective public policies and programs to prevent the consumption of UPPs among children. It is important to promote the replacement of UPPs with ‘real food’ with health protective effects [68] and separate them from the notion that they are a way of expressing affection and other good feelings. This effort should be global and a call for attention directed not only to parents, grandparents, and siblings but to all members of society, including teachers, health professionals, and decision-makers.

In short, culture is not static and is constantly being built into interaction with others, with corporate commercial strategies, and/or with government efforts to promote health. For these reasons, countries should reinforce public policies that regulate the food environment in order to protect children and control UPP availability and access. This includes providing information on the healthfulness of these products (through the regulation of marketing and labeling) without forgetting innovative strategies such as mHealth and its use of new technologies to disseminate evidence-based information that is free of conflicts of interest. In effect, removing UPPs from the day-to-day food culture in general and from child-feeding practices requires stronger public policies to change social norms, which currently favor children’s consumption of UPPs, as has previously been performed with tobacco. 

## Figures and Tables

**Table 1:** Main sociodemographic characteristics of PCs.

Variable		Yucatan	Morelos
		Mean (SD)	Mean (SD)
Age		34.6 (2.6)	32.2 (1.5)
		[*n* = 12]	[*n* = 12]
	Primary school	5	4
	Secondary	2	7
School years	High School	4	1
	Technical career	1	0
		*n* [12]	*n* [12]
Civil status	Married	12	6
	Single	-	1
	Cohabitation	-	5
		Mean	Mean
Number of children		3.2 (0.40)	3.6 (0.35)
Number of people living in the household		5.4 (0.5)	6.2 (0.5)

## Supplementary Materials

The following supporting information can be downloaded at: https://www.mdpi.com/article/10.3390/nu15061317/s1, Figure S1: Coding Tree; Table S1: The sociodemographic information NUTRES.
