# Supplementary material for: Challenges around Child-Feeding Practices with ‘Comida Chatarra’: A Qualitative Study to Understand the Role of Sociocultural Factors in Caregiver Feeding Decisions
Source: Nutrients. 2023 Mar 7;15(6):1317. doi: 10.3390/nu15061317 (PMC10054330; doi:10.3390/nu15061317)
Supplement: Supplementary file 1 [file nutrients-15-01317-s001.zip › Figure S1. Coding Tree.pdf]

CODE	DEFINITION
PCs’experience with UPPS.	It includes all the information on ‘UPPs’ (ETIC viewpoint) and ‘junk (EMIC viewpoint) related to attitude, knowledge, behavior, source of in
1. Knowledge	Set of knowledge that PCs have about junk food
Junk food notion	General ideas that PCs associate with junk food
Nomenclature	Ethnocategory or local terminology to refer to "junk food"
Type of Knowledge	Type of knowledge mobilized by PCs in relation to junk food (sanitary, popular, based on their own experience, etc.).
Health damage	Set of general damages associated with the consumption of UPPs mentioned by the PCs.
Junk food	Knowledge related to the composition of these products.
Children damage	Set of children’s damages associated with the consumption of UPPs mentioned by the CPs.
**2. Family dynamics**	Set of information related to the dynamic families around junk food.
Supply and purchasing	Información que describe abastecimiento de la famlia de los UPPs y de la junk food
Circumstances of consumption	Información relativas a todas las circonstancias de consumo de la junk food en familia (fiestas, paseos familares, etc)
Feelings and emotions	Feelings and emotions associated with the consumption of junk food.
**3. Feeding practices y behavior**	Set of information related to feeding practices with UPPs (ETIC) and junk food (EMIC).
Circumstances of consumption	Description of the circumstances and moments of consumption of UPPs and junk food by children.
Type of products consumed	Universe of products consumed by children (ETIC and EMIC) (including the most consumed)
Consumption frequency	Number of times children eating "junk food" during a period of time.
justification	Justification for why the child consumes "junk" food (child's request, PC initiative, etc.)
Determinants of the UPPs of the feeding choice	Set of elements that facilitate, according to the PCs, the UPPs feeding choice (offer, children taste, convenience, price)-

Providers	Person that provides or supplies the junk food.
**4. PCs' attitudes**	Set of PCs' opinion toward junk food.
Appreciation toward junk food	Set of opinion favorable and unfavorable of PCs toward UPPs in general.
Junk food type	Opinion of the PCs according to type of junk food.
Justification	Mobilized arguments to justify the position of the Pcs toward UPPs.
Control	Set of information linked to strategies developed by the CPs to reduce the consumption of junk food by children.
Award	Set of information related to the children's prize with junk food.
**5. Children's attitudes toward junk food**	Set of information that addresses the attitude of children towards junk (do they ask for it? Do they like it?).
**6. Information**	Set of information related to junk food that reached the PCs.
Source of information	PCs' sources of information about junk food.
Messages	Main messages issued by different sources about PUPs.
Appreciations	PCs' opinion about these messages.
**7. Social norms**	Set of information mentioned by PCs to identify the social norm related to the consumption of UPPs by adult and child population.
Consumption in the areas	Information related to UPPs consumption in the area by children and adults (the consumption is widespread?)
CPs' appreciations toward junk food consumption.	Opinions of PCs about the extension and generalization of consumption within the community (adult and child population)
