# Supplementary material for: Challenges around Child-Feeding Practices with ‘Comida Chatarra’: A Qualitative Study to Understand the Role of Sociocultural Factors in Caregiver Feeding Decisions
Source: Nutrients. 2023 Mar 7;15(6):1317. doi: 10.3390/nu15061317 (PMC10054330; doi:10.3390/nu15061317)
Supplement: Supplementary file 1 [file nutrients-15-01317-s001.zip › Table S1 The sociodemographic information NUTRES.pdf]

Table S1. The sociodemographic information of the 24 participants

	ID	State and Area	Sex	Age	Civil status	School years	Number of children	Child age	Number of people living at home
	**#1**	Sate 1. Rural area	M	30	Married	High School	2	4	4
	**#2**	Sate 1. Rural area	M	37	Married	Primary school	3	4	5
	**#3**	Sate 1. Rural area	M	30	Married	Secondary	3	2	5
	**#4**	Sate 1. Rural area	M	26	Married	Secondary	2	2	4
	**#5**	Sate 1. Rural area	M	36	Married	Primary school	3	4	4
	**#6**	Sate 1. Rural area	M	29	Married	High School	4	3	7
	**#7**	State 1. Urbano area	M	45	Married	Primary school	3	4	6
	**#8**	State 1. Urbano area	M	55	Married	Technical career	3	2	6
	**#9**	State 1. Urbano area	M	39	Married	Primary school	4	3	5
	**#10**	State 1. Urbano area	M	37	Married	Primary school	7	3	10
	**#11**	State 1. Urbano area	M	24	Married	High School	2	3	5
	**#12**	State 1. Urbano area	M	27	Married	High School	2	3	4
	**#13**	Sate 2. Rural area	M	27	Cohabitation	Secondary	3	3	5
	**#14**	Sate 2. Rural area	M	35	Cohabitation	Primary school	6	3.5	8
	**#15**	Sate 2. Rural area	M	34	Cohabitation	Secondary	5	4	9

	**#16**	Sate 2. Rural area	M	36	Married	Primary school	5	5	SR
	**#17**	Sate 2. Rural area	M	34	Cohabitation	Secondary	4	3.4	6
	**#18**	Sate 2. Rural area	M	41	Married	High School	3	2	6
	**#19**	State 2. Urbano area	M	31	Married	Secondary	4	3	6
	**#20**	State 2. Urbano area	M	36	Married	Primary school	2	3	3
	**#21**	State 2. Urbano area	M	36	Married	Secondary	3	3	5
	**#22**	State 2. Urbano area	M	26	Married	Secondary	3	4	5
	**#23**	State 2. Urbano area	M	28	Cohabitation	Primary school	4	2	9
	**#24**	State 2. Urbano area	M	23	Single	Secondary	2	2	6
